# The Role of Stem Cells in Peripheral Nerve Regeneration: A Narrative Review

**DOI:** 10.7759/cureus.91459

**Published:** 2025-09-02

**Authors:** Deepa G, Shrikrishna B H

**Affiliations:** 1 Anatomy, All India Institute of Medical Sciences, Bibinagar, Hyderabad, IND; 2 Otorhinolaryngology - Head and Neck Surgery, All India Institute of Medical Sciences, Bibinagar, Hyderabad, IND

**Keywords:** clinical trials, mesenchymal stem cells, nerve regeneration, peripheral nerves, stem cells

## Abstract

Stem cell therapies are gaining attention as a potential strategy for promoting recovery in peripheral nerve injuries. These injuries, particularly in complex anatomical regions, often lead to long-term functional deficits. There is a pressing need to explore novel therapeutic approaches. Experimental studies have shown that various stem cell types possess regenerative capabilities. This review synthesizes existing experimental and clinical evidence, highlighting the types of stem cells investigated, their mechanisms of action, and reported functional outcomes. Among them, adipose-derived and bone marrow-derived mesenchymal stem cells have demonstrated consistent improvements in nerve function and structural repair. Other cell types, including hair follicle and dental pulp stem cells, have also shown promising outcomes in preclinical models. Clinical studies remain limited, with only a few reporting partial recovery and minimal adverse effects. However, the overall quality of clinical evidence is low, limiting the strength of conclusions. The current evidence suggests potential, but it remains insufficient for broad clinical application. The review identifies key knowledge gaps, such as limited large-scale human trials, and underscores the need for high-quality research to determine the safety, efficacy, and feasibility of stem cell-based therapies for peripheral nerve repair. Further high-quality human trials are urgently needed to establish the safety and efficacy of these therapies.

## Introduction and background

Peripheral nerve injuries cause significant long-term disability, impairing motor, sensory, and autonomic functions, and severely affecting quality of life. They may result from trauma, surgical interventions, or disease, and their repair remains a major clinical challenge [1]. Conventional methods such as end-to-end neurorrhaphy, nerve grafting, and nerve conduits are standard but often fail to achieve full functional recovery due to restricted axonal regeneration and incomplete target muscle reinnervation [2].

Among various regenerative approaches explored in recent years, stem cell therapy has gained particular attention for its distinct biological advantages. Stem cells can promote nerve repair by releasing neurotrophic factors, modulating inflammation, and differentiating into Schwann-like cells that support axonal guidance [3]. Multiple stem cell types, including bone marrow-derived mesenchymal stem cells [4], adipose-derived stem cells [5], and hair follicle-associated stem cells [6], have shown promising results in preclinical models by enhancing axonal sprouting and remyelination. Compared to other regenerative strategies, stem cell-based therapies offer the unique potential for both structural and functional restoration through multiple synergistic mechanisms.

However, despite encouraging experimental data, translation into routine clinical use remains limited, with variability in cell sources, administration techniques, and outcome measures posing challenges [7]. A detailed review focused specifically on stem cell therapy is therefore warranted to consolidate current evidence, highlight translational barriers, and inform future research directions [8].

## Review

Methods

Eligibility Criteria

Included studies were in vivo experimental or clinical studies investigating peripheral nerve injuries, specifically in the context of stem cell therapy. Interventions had to involve stem cells, either alone or combined with adjunct therapies. Outcomes had to include functional recovery measures. In vitro-only studies and those lacking appropriate controls were excluded. 

Search Strategy

A search was conducted in PubMed using the Boolean operator string: ((stem cell therapy[Title/Abstract]) AND (peripheral nerve[Title/Abstract])) AND (regeneration[Title/Abstract]). This search yielded 50 results initially. The keywords were chosen after preliminary exploratory searches and refined using Boolean operators to maximize relevant retrieval while minimizing irrelevant hits. Automatic filters were then applied sequentially to refine the search: studies published in the last 10 years (n=42), free full-text availability (n=27), English language restriction (n=27), and exclusion of review articles (n=17). After applying all filters and removing duplicates, 17 articles were screened for eligibility.

Filters were applied to ensure the retrieval of recent, accessible, and relevant primary research. The last 10 years limit ensured contemporary evidence; free full-text and English language restrictions enabled full data extraction, and excluding reviews focused the analysis on original studies, improving validity and reproducibility. The screening process was performed independently by both reviewers. The date of the search was May 10, 2025. 

Studies were included based on predefined eligibility criteria that ensured relevance and methodological rigor. These criteria required a focus specifically on peripheral nerve injuries rather than central nervous system damage, and the inclusion of a stem cell-based intervention, either alone or in combination with adjunctive therapies. Eligible studies employed in vivo models, including both animal experiments and human clinical or observational research. Additionally, studies were required to report quantitative functional outcome measures related to nerve regeneration. Finally, only studies with adequate methodological clarity, including the presence of comparator groups or controls, were considered for inclusion.

Studies were excluded if they were limited to in vitro experiments, did not utilize stem cells as the primary intervention, failed to report functional outcomes related to nerve regeneration, or did not meet basic quality thresholds in study design.

After full-text screening, 14 studies met all eligibility criteria and were included in the final synthesis. Three studies were excluded due to incomplete outcome reporting or inappropriate model systems.

Data Collection

The following data were extracted: author, year, country, study design, population/species, stem cell type and source, intervention method, comparator (if present), outcomes, key findings, and reported adverse events.

Risk of Bias 

Risk of bias was assessed using the Newcastle-Ottawa Scale. Bias domains included selection, performance, detection, attrition, and reporting biases. Although this review is narrative in design, the Newcastle-Ottawa Scale was employed to systematically assess the quality and risk of bias of the included observational studies, owing to its established validity and widespread use. 

Data Synthesis

Due to heterogeneity in study designs and outcome metrics, a narrative synthesis was performed. Data were summarized in tables, categorized by stem cell type and study type (experimental vs. clinical).

Results

**Figure 1:**
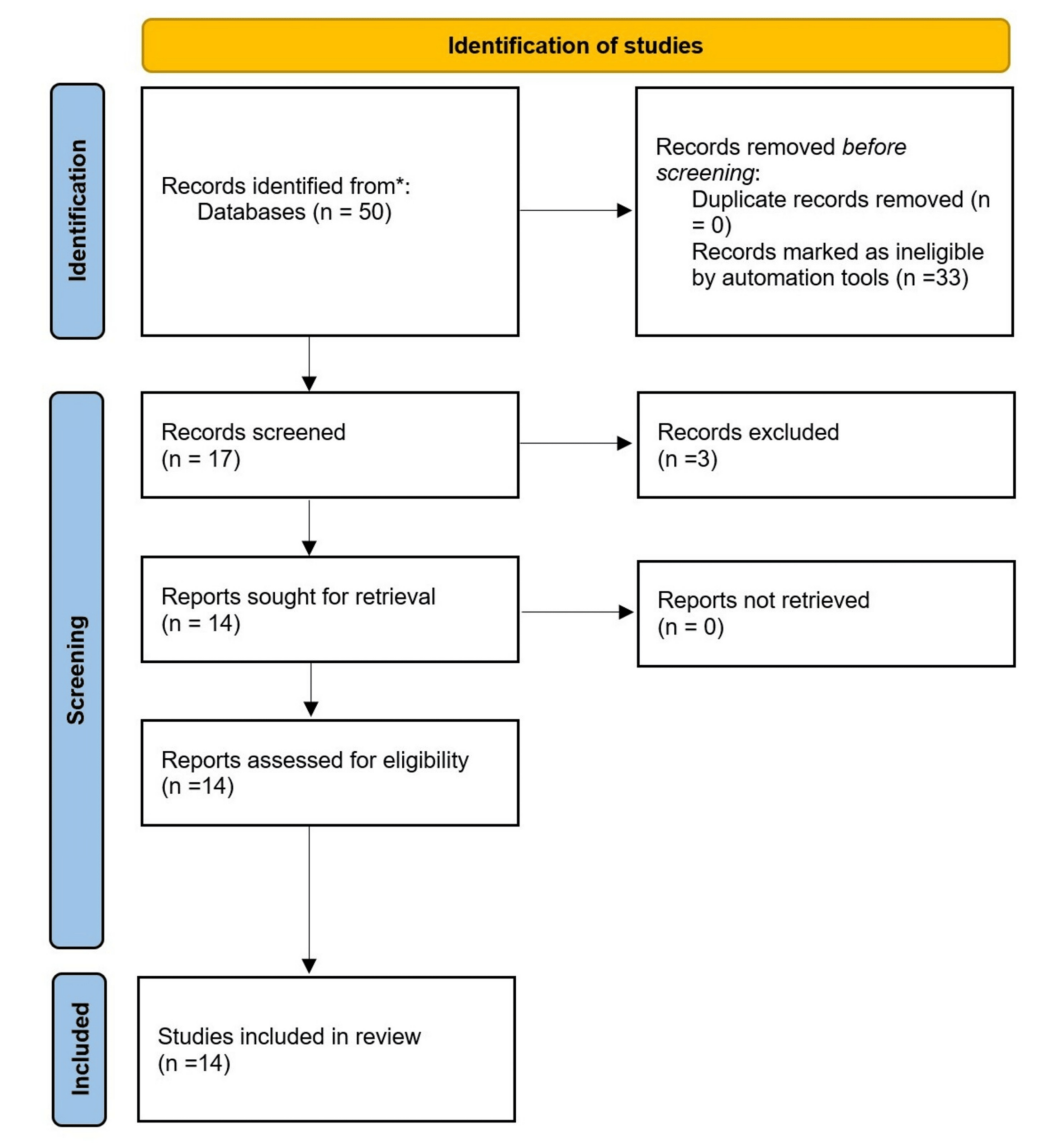
PRISMA Flow Diagram PRISMA: Preferred Reporting Items for Systematic Reviews and Meta-Analyses

The study selection process is illustrated in the Preferred Reporting Items for Systematic Reviews and Meta-Analyses (PRISMA) flow diagram in Figure 1.

The characteristics and key findings of the included studies are summarized in the data extraction table (Table 1), and include information such as Author(s), Year, Country, Study Design, Population, Exposure Type, Comparator, Outcomes, and Key Findings.

**Table 1:** Summary of data from included studies RCT: randomized controlled trial; SFI: Sciatic Functional Index; EMG: electromyography; CMAP: compound muscle action potential; BBB: Basso–Beattie–Bresnahan locomotor rating scale

Author(s), year	Country	Study Design	Population	Exposure Type	Comparator	Outcomes	Key Findings
Bingham et al., 2024 [1]	United States	Experimental animal study	Rats (n=9 per group)	Bone marrow-derived mesenchymal stem cells	Vehicle control	SFI, Sensory function	SFI improved from -77 to -34 at 8 weeks (P < 0.05)
Schweizer et al., 2020 [2]	United States	Experimental animal study, RCT	Rats (n=10-12 per group)	Adipose-derived stem cells	Repair/cut control	Static Sciatic Index, swim test, toe spread factor	Superior static indices (P < 0.0001 at 6 weeks)
Huang et al., 2019 [3]	Taiwan	Experimental animal study, RCT	Rats	Multipotent vascular stem cells	Control group	Compound muscle action potential, Schwann cell recruitment	CMAP increased (p<0.01) at 1 month
Hejazian et al., 2022 [4]	Iran	Experimental animal study	Rats	Hair follicle stem cells	Control group	Electromyography, histology	EMG amplitude (P=0.001), latency (P=0.012) at 8 weeks
Hei et al., 2023 [5]	China	Experimental animal study, in vitro	Rats	Human dental pulp stem cells	Knockdown group	Sensory test, axon counts	Better sensory scores (P<0.05) at 1-3 weeks
Zhang et al., 2024 [6]	China	Experimental animal study, in vitro	Rats	Adipose mesenchymal stem cells	Control groups	SFI, CMAP	Superior outcomes with chitosan/magnetic targeting at 12 weeks
Nam et al., 2023 [7]	South Korea	Experimental animal study	C22 mice (CMT model)	Tonsil-derived mesenchymal stem cells	Control group	SFI, nerve conduction velocity, CMAP	High-dose group showed significant improvement at 4-12 weeks
Gao et al., 2024 [8]	China	Experimental animal study	Rats	Bone marrow-derived mesenchymal stem cells	Control group	Histology	Increased myelination and fiber density at 28 days
Hashimoto et al., 2016 [9]	Japan	Experimental animal study	Mice	Skeletal muscle-derived multipotent stem cells	Control group	Tetanic tension, muscle mass	36-49% tetanic tension recovery at 4-18 weeks
Xiao et al., 2024 [10]	China	Observational study, in vitro	Humans	Adipose-derived mesenchymal stem cells (nanofat)	Historical controls	Two-point discrimination, Semmes-Weinstein	Significant sensory improvement at 2 months
Du et al., 2018 [11]	China	Experimental animal study, in vitro	Rats (n=60)	Human neural crest stem cells	Control groups	Stand time	72-77% stand time recovery with electrical stimulation at 6-12 weeks
Rached et al., 2025 [12]	Brazil	Clinical case report	Human (n=1)	Adipose-derived mesenchymal stem cells	Pre-treatment status	Electromyography, torque, power	Full EMG recovery, 55% torque increase at 2 months
Maldonado et al., 2025 [13]	United States	Clinical case report, observational	Humans (n=5)	Porcine, adipose, fetal stem cells	Pre-treatment status	Electrophysiology, clinical assessment	No objective improvement reported
Lee et al., 2021 [14]	South Korea	Experimental animal study	Rats	Peripheral nerve-derived stem cells	Control group	Basso, Beattie, and Bresnahan score	BBB score 10.2±0.95 at 8 weeks with spheroids

The risk of bias for each included study was assessed across key domains and is summarized in Table 2.

**Table 2:** Risk of bias assessment N/A: not applicable

Study	Randomization	Allocation Concealment	Blinding	Incomplete Data	Selective Reporting	Other Bias	Overall Risk
Bingham et al.,2024 [1]	Unclear	Unclear	Unclear	Low	Unclear	Unclear	Moderate
Schweizer et al., 2020 [2]	Low	Unclear	Unclear	Low	Low	Low	Low-Moderate
Huang et al., 2019 [3]	Low	Unclear	Unclear	Unclear	Unclear	Unclear	Moderate
Hejazian et al., 2022 [4]	Unclear	Unclear	Unclear	Low	Unclear	Unclear	Moderate
Hei et al., 2023 [5]	Unclear	Unclear	Unclear	Low	Unclear	Unclear	Moderate
Zhang et al., 2024 [6]	Unclear	Unclear	Unclear	Low	Unclear	Unclear	Moderate
Nam et al., 2023 [7]	Unclear	Unclear	Unclear	Low	Unclear	Unclear	Moderate
Gao et al., 2024 [8]	Unclear	Unclear	Unclear	Low	Unclear	Unclear	Moderate
Hashimoto et al., 2016 [9]	Unclear	Unclear	Unclear	Low	Unclear	Unclear	Moderate
Xiao et al., 2024 [10]	N/A	N/A	N/A	Unclear	Unclear	High	High
Du et al., 2018 [11]	Unclear	Unclear	Unclear	Low	Unclear	Unclear	Moderate
Rached et al., 2025 [12]	N/A	N/A	N/A	Low	Unclear	High	High
Maldonado et al., 2025 [13]	N/A	N/A	N/A	Unclear	Unclear	High	High
Lee et al., 2021 [14]	Unclear	Unclear	Unclear	Low	Unclear	Unclear	Moderate

Study Characteristics Summary 

A total of 14 studies were included. Of these, 11 were experimental animal studies [1-9,11,14], one was an observational human study [10], and two were clinical case reports [12,13]. Commonly studied nerves included the sciatic nerve (rats/mice), the mental nerve, and the peripheral nerves in clinical settings. Stem cell types included adipose-derived, bone marrow-derived, hair follicle, human dental pulp, neural crest, tonsil-derived (Schwann-like), and skeletal muscle-derived cells.

Discussion

This review synthesized evidence from 14 studies investigating the effectiveness of various stem cell types in peripheral nerve regeneration, encompassing both experimental animal models and clinical applications. The findings demonstrate that multiple stem cell types show therapeutic potential for peripheral nerve repair, though with considerable heterogeneity in outcomes and methodological approaches. Bone marrow-derived mesenchymal stem cells consistently demonstrated functional improvements across studies [9,10], while adipose-derived stem cells showed both experimental efficacy [2,6] and promising clinical results in individual cases. Novel stem cell sources, including hair follicle stem cells [4], dental pulp stem cells [5], and neural crest stem cells [11], also showed significant therapeutic potential, suggesting that the regenerative capacity is not limited to traditional mesenchymal stem cell sources.

Comparative Effectiveness Across Stem Cell Types

The comparative analysis reveals that mesenchymal stem cells from various sources maintain therapeutic efficacy but with distinct advantages depending on the specific application. Adipose-derived stem cells demonstrated particular promise due to their accessibility, minimal donor site morbidity, and consistent functional outcomes [2,6,12]. The ability to enhance their therapeutic potential through combination with biomaterial scaffolds and targeting systems [6] suggests opportunities for optimization that may not be as readily available with other stem cell types. Bone marrow-derived mesenchymal stem cells, while requiring more invasive harvesting procedures, showed robust and sustained functional improvements [1,8], particularly when delivered via appropriate scaffold systems that enhance cell retention and survival.

Specialized stem cell types offer unique advantages that may be particularly relevant for specific clinical scenarios. Hair follicle stem cells [4] and dental pulp stem cells [5] demonstrated rapid therapeutic effects with measurable improvements within weeks of administration, potentially offering advantages in acute injury settings. Neural crest stem cells [11] and peripheral nerve-derived stem cells showed particular affinity for neural regeneration, possibly reflecting their developmental lineage relationships with peripheral nervous system components. Tonsil-derived mesenchymal stem cells differentiated to Schwann cell-like phenotype [7] represent an innovative approach that combines stem cell therapy with directed differentiation, potentially enhancing the specificity of therapeutic effects.

Mechanisms of Therapeutic Action

The included studies highlighted multiple mechanisms through which stem cells promote peripheral nerve regeneration, though the mechanistic investigation was not consistently performed across all studies. The primary mechanisms identified include secretion of neurotrophic factors that support axonal growth and survival, direct differentiation into neural lineage cells, including Schwann cells that provide structural and trophic support for regenerating axons, modulation of inflammatory responses that can either promote or inhibit regenerative processes, and enhancement of the local regenerative microenvironment through paracrine signaling. Several studies specifically documented increased Schwann cell recruitment [3], enhanced myelination [4,8], and improved axonal counts [5], suggesting that both direct cellular replacement and indirect trophic support contribute to therapeutic efficacy.

The combination of stem cells with adjuvant therapies appeared to enhance therapeutic outcomes through synergistic mechanisms. Electrical stimulation combined with neural crest stem cells [11] achieved superior functional recovery compared to stem cells alone, possibly through enhanced axonal sprouting and target reinnervation. Similarly, the combination of stem cells with biomaterial scaffolds [6,8] provided sustained cell delivery and created favorable microenvironments for regeneration. The use of magnetic targeting systems [6] represents an innovative approach to enhance cell retention at injury sites, potentially improving the efficiency of stem cell delivery and reducing the number of cells required for therapeutic effect.

Clinical Translation Challenges and Opportunities

The limited clinical evidence reveals significant challenges in translating promising experimental results to human applications. The stark contrast between the successful case report [12] and the negative case series [13] emphasizes the importance of multiple factors, including stem cell source and preparation, delivery method, patient selection, timing of intervention, and outcome assessment. The successful clinical case utilized autologous adipose-derived mesenchymal stem cells delivered via ultrasound-guided local injection, while the unsuccessful series used heterologous cell sources with various delivery methods, suggesting that autologous cells and precise delivery may be critical for clinical success [14]. The key factors that hinder clinical translation are regulatory hurdles, inconsistent clinical outcomes, and unresolved long-term safety issues.

Stem cell factor (SCF) is a crucial cytokine that binds to the c-kit receptor on stem and progenitor cells, playing a vital role in their survival, proliferation, and differentiation, especially within the hematopoietic system. SCF is produced by various cells, including endothelial cells and fibroblasts, and is essential for maintaining the bone marrow microenvironment that supports hematopoietic stem cells (HSCs) and their progenitors, ensuring the continuous production of blood cells and the health of the hematopoietic niche [15-17]. In the placenta and other fetal tissues, SCF is key for the development and self-renewal of HSCs, helping balance cell survival and death, particularly under hypoxic conditions, by regulating autophagy and apoptosis [17].

SCF acts through its receptor c-Kit, a tyrosine kinase on stem and progenitor cells, to regulate survival, proliferation, and differentiation. SCF binding activates key pathways, PI3K/AKT, RAS/MAPK, and JAK/STAT, that work in concert with Wnt, Notch, and TGF-β signaling. This coordinated activity maintains stem cell function and tissue homeostasis, supporting its therapeutic relevance [18,19,20].

Stem cells play a promising role in peripheral nerve regeneration by providing a source of cells that can support and enhance the repair of damaged nerves. Mesenchymal stem cells, particularly those derived from adipose tissue, are highlighted for their accessibility, low immunogenicity, and ability to differentiate into Schwann-like cells, which are crucial for nerve repair and remyelination [21-24]. These stem cells secrete neurotrophic and angiogenic factors, modulate inflammation, and create a supportive microenvironment that promotes axonal growth and functional recovery [25,26]. Incorporating stem cells into bioengineered nerve conduits or scaffolds further boosts regeneration, offering an alternative to traditional autografts and reducing donor site morbidity [27-29]. Preclinical and early clinical studies demonstrate that stem cell therapies can enhance nerve regeneration, remyelination, and revascularization, although challenges remain regarding standardization, immune response, and long-term safety [22,30]. Overall, stem cell-based interventions represent a significant advance in regenerative medicine for peripheral nerve injuries, with ongoing research aimed at optimizing protocols and translating these therapies into routine clinical practice. 

Several factors may contribute to the translational gap between experimental and clinical outcomes. Animal models typically involve acute, standardized injuries in young, healthy subjects, while clinical cases often involve chronic injuries in patients with comorbidities that may impair regenerative capacity. The timing of intervention appears critical, with experimental studies typically administering stem cells immediately or within days of injury, while clinical applications often occur weeks to months after initial injury, when the regenerative window may be compromised. Additionally, the heterogeneity in stem cell preparation, dosing, and delivery methods across clinical studies makes it difficult to establish optimal treatment protocols.

Methodological Considerations and Limitations

Several methodological limitations affect the interpretation and generalizability of the current evidence. The majority of experimental studies lacked adequate randomization and blinding procedures, with risk of bias assessment revealing moderate to high risk across most studies. Sample sizes were frequently small and not consistently reported, limiting statistical power and generalizability of findings. The heterogeneity in outcome measures across studies prevents meaningful quantitative synthesis and makes direct comparisons between interventions challenging. Furthermore, the follow-up periods varied substantially, from weeks to months, making it difficult to assess the long-term efficacy and safety of stem cell interventions. A key limitation is the scarcity of human studies and the predominance of case reports, coupled with a high risk of bias across most studies, which limits the generalizability of the findings.

The clinical studies represent the earliest stages of clinical investigation, consisting primarily of case reports and small case series without appropriate controls. This level of evidence, while providing proof-of-concept data, is insufficient to establish clinical efficacy or safety. The lack of standardized outcome measures in clinical studies further complicates the assessment of therapeutic benefit. Additionally, the absence of systematic safety reporting across both experimental and clinical studies represents a significant gap that must be addressed before widespread clinical implementation can be considered.

The purpose of this review was to critically evaluate the role of stem cells in peripheral nerve regeneration, addressing the current lack of consolidated understanding in this evolving field. Our synthesis indicates that, despite differences in stem cell sources, delivery methods, and experimental models, a consistent trend toward enhanced axonal regeneration, remyelination, and functional recovery is evident. This directly fulfills the objective of identifying and integrating diverse findings into a coherent evidence-based perspective. The observed variability in study outcomes highlights the necessity for standardized protocols and long-term clinical trials, while the overall positive direction of results supports the potential of stem cell-based therapies as a promising adjunct in peripheral nerve repair.

Future Research Directions

Several research priorities emerge from this review that could advance the field toward clinical translation. Large-scale, well-designed randomized controlled trials are urgently needed to establish the clinical efficacy and safety of stem cell therapy for peripheral nerve regeneration. These studies should incorporate standardized outcome measures, appropriate control groups, adequate follow-up periods, and systematic safety assessment. Comparative effectiveness research directly comparing different stem cell types, delivery methods, and combination therapies could help identify optimal treatment strategies for specific clinical scenarios.

Mechanistic research remains important to understand the cellular and molecular basis of stem cell-mediated nerve regeneration, which could inform optimization of therapeutic approaches. Investigation of factors that influence clinical translation, including patient selection criteria, timing of intervention, and methods to enhance stem cell survival and integration, represents critical research priorities. Additionally, the development of standardized protocols for stem cell isolation, characterization, expansion, and delivery could improve the consistency and reproducibility of therapeutic outcomes across different clinical centers.

The development of combination therapies that integrate stem cells with biomaterial scaffolds, growth factors, electrical stimulation, or other regenerative approaches shows particular promise and warrants systematic investigation. Furthermore, research into biomarkers that could predict therapeutic responses and guide personalized treatment approaches could enhance clinical outcomes. Finally, long-term follow-up studies are needed to assess the durability of therapeutic benefits and identify any delayed adverse effects of stem cell interventions.

## Conclusions

Stem cell therapy shows encouraging results for peripheral nerve regeneration, especially with bone marrow- and adipose-derived mesenchymal stem cells. Clinical evidence is still limited, and further large, well-designed trials are needed to confirm safety and effectiveness. Until then, its use should remain within controlled research settings. The current evidence provides a foundation for continued research and development in this promising therapeutic area while highlighting the need for rigorous clinical investigation to establish safety and efficacy before widespread clinical implementation. The potential for stem cell therapy to address the significant unmet clinical need in peripheral nerve regeneration justifies continued investment in high-quality research to advance this field toward clinical reality. Future research should focus on standardized outcome measures, defined cell preparation protocols, long-term safety assessments, and addressing key study design gaps before clinical implementation. 
